# Current News

**Published:** 1896-10

**Authors:** 


### Current News.

### NORTHEASTERN DENTAL ASSOCIATION.

The annual meeting of the Northeastern Dental Association will be held in Springfield, Mass., October 21, 22, 23, 1896. An interesting meeting is promised, and a large attendance is desired. Please mark off the dates on your appointment books *now* and make an effort to be present. Programmes will be sent in due time.

Edgar O. Kinsman, D.D.S.,
*Secretary.*

Cambridge, Mass., September 19, 1896.

